# Tumor neoantigens: Novel strategies for application of cancer immunotherapy

**DOI:** 10.32604/or.2023.029924

**Published:** 2023-06-27

**Authors:** HANYANG GUAN, YUE WU, LU LI, YABING YANG, SHENGHUI QIU, ZHAN ZHAO, XIAODONG CHU, JIASHUAI HE, ZUYANG CHEN, YIRAN ZHANG, HUI DING, JINGHUA PAN, YUNLONG PAN

**Affiliations:** 1 Department of General Surgery, The First Affiliated Hospital of Jinan University, Guangzhou, 510632, China; 2 Department of Gynecology, Women’s Hospital of Nanjing Medical University, Nanjing Maternity and Child Health Care Hospital, Nanjing, 210004, China; 3 Department of Gastroenterology, The First Affiliated Hospital of Jinan University, Guangzhou, 510630, China

**Keywords:** Immunotherapy, Tumor vaccine, Adoptive T cell therapy, Chimeric antigen receptor

## Abstract

Neoantigen-targeted immunotherapy is a rapidly advancing field that holds great promise for treating cancer. The recognition of antigens by immune cells is a crucial step in tumor-specific killing, and neoantigens generated by mutations in cancer cells possess high immunogenicity and are selectively expressed in tumor cells, making them an attractive therapeutic target. Currently, neoantigens find utility in various domains, primarily in the realm of neoantigen vaccines such as DC vaccines, nucleic acid vaccines, and synthetic long peptide vaccines. Additionally, they hold promise in adoptive cell therapy, encompassing tumor-infiltrating cells, T cell receptors, and chimeric antigen receptors which are expressed by genetically modified T cells. In this review, we summarized recent progress in the clinical use of tumor vaccines and adoptive cell therapy targeting neoantigens, discussed the potential of neoantigen burden as an immune checkpoint in clinical settings. With the aid of state-of-the-art sequencing and bioinformatics technologies, together with significant advancements in artificial intelligence, we anticipated that neoantigens will be fully exploited for personalized tumor immunotherapy, from screening to clinical application.

## Introduction

Malignant tumors pose a significant hurdle to extend human lifespan, but modern medicine has relied on surgical removal, chemotherapy, and radiation therapy to combat them. Nonetheless, tumor immunotherapy has made impressive strides due to advancements in technology and an immune system [1]. Recently, clinical trials have intensified in the field of tumor neoantigens, which represent a new approach to immunotherapy, distinct from traditional immune checkpoint inhibitor therapies. Unlike tumor-associated antigens (TAAs), tumor-specific antigens (TSAs), also known as neoantigens, are exclusive to tumors and do not exist in healthy human tissues or organs. These unique peptides result from complex mutations that occur within the tumor’s environment during growth, influenced by physical, chemical, and biological factors. Classification by the mechanism of neoantigen generation, reveals that somatic genomic alterations predominantly contribute to the production of tumor neoantigens, such as single-nucleotide variants (SNVs). This process also extends to transcriptomic and proteomic variants, including alternative splicing of transcripts and post-translational modifications (PTMs) [2]. Tumor-specific neoantigens generated by genetic changes can activate high-affinity T cells due to their inherent tumor specificity and immunogenicity. As such, they serve as effective natural targets for tumor immunotherapy [3,4]. This review provides an overview on the clinical applications in the development of neoantigen-based tumor vaccines [5–8], adoptive cell therapy (ACT) [9–11], and exploring the correlation between tumor neoantigen burden and immunotherapy efficacy [12–14].

## Neoantigen-Based Therapeutic Cancer Vaccines

### Dendritic cell vaccines

DC vaccines possess potent immune-stimulatory properties. Through the combination of relevant antigens with antibodies or ligands *in vitro*, DCs can effectively target receptors specific to their kinds, leading to the phagocytosis, processing, and presentation of antigens to immune cells, including T cells [15]. This process promotes the induction of tumor-specific CD4+ T cells and cytotoxic T cells (CTLs) within the human body, culminating in an anti-tumor effect mediated by immune cells.

**Figure 1:**
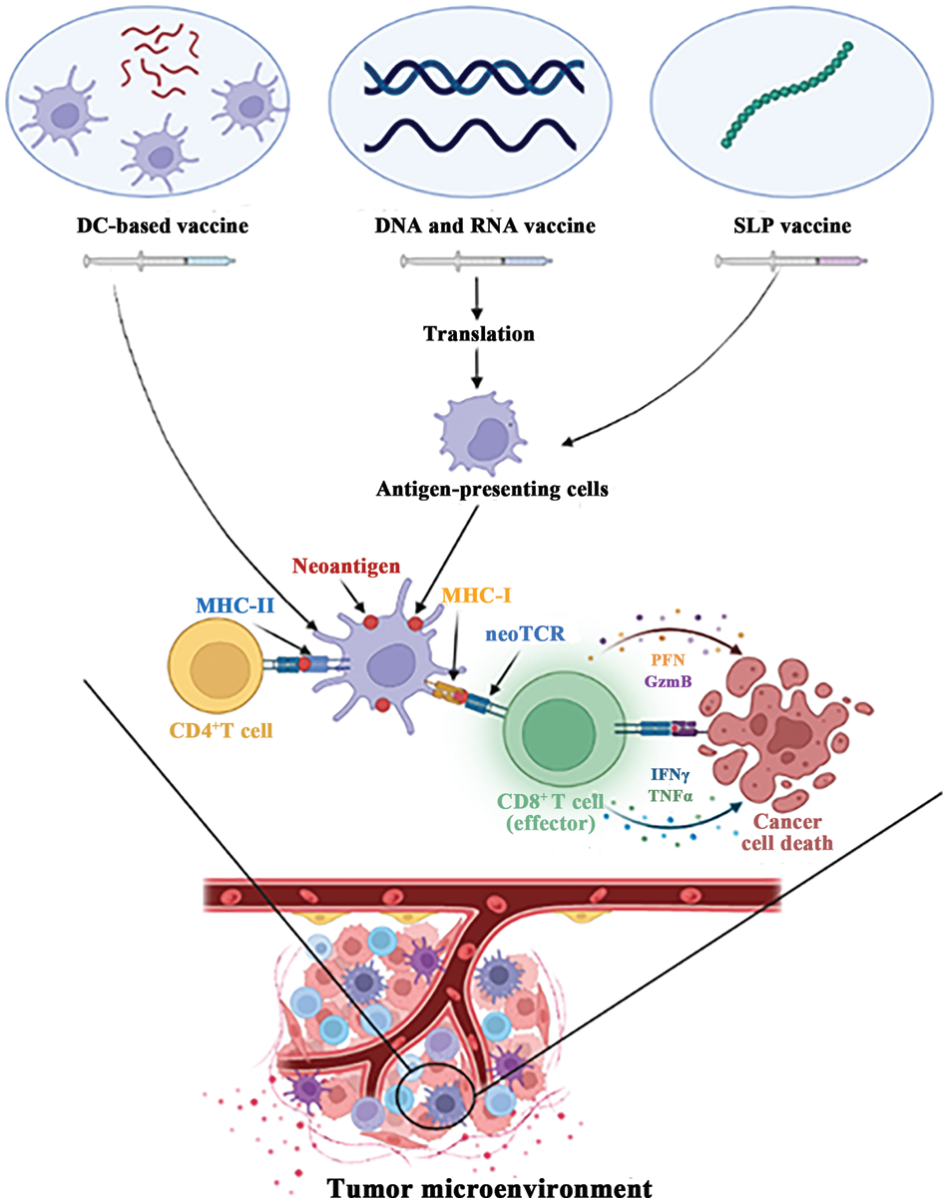
Major types of neoantigen vaccine. In tumor microenvironment, neoantigen vaccines can activate CD8+ T cells and CD4+ T cells to kill tumor cells.

To load the neoantigen DC vaccine, the peptide corresponding to the neoantigen is co-cultivated with the vaccine and introduced to the body through various routes (Fig. 1). The clinical response rate and safety of this approach has been effectively guaranteed [16]. In a phase II clinical trial for liver cancer [17], the use of personalized neoantigen-loaded DC vaccine, and neoantigen-activated T cell therapy as adjuvant treatments, significantly improved the recurrence rate of patients undergoing radical resection or radiofrequency ablation for HCC. Furthermore, disease free survival (DFS) was significantly prolonged. In a prospective study of patients with advanced lung cancer [18], the neoantigen DC vaccine was deemed safe and tolerable, displaying no adverse effects on immune checkpoint inhibitor (ICI) therapy. Through nature-inspired techniques, the development of DC vaccines has proven to be a promising avenue in cancer treatment.

The latest generation of DC vaccines derives specific DC subpopulations from natural sources found in patients, leading to superior functionality and reduced costs [19]. This approach not only proves to be safe and feasible, but also significantly enhances efficacy [20,21]. For instance, the vaccination of melanoma patients with naturally occurring plasmacytoid dendritic cells (pDCs) resulted in a unique IFN signature after each vaccination, indicating an improvement in therapeutic outcomes [20]. This increased curative effect may be attributed to the superior immune efficacy of the particular DC subpopulation over others [21]. While clinical trials have successfully demonstrated the safety and effectiveness of DC vaccines, their efficacy in real-world practice has yet to be fully validated, and issues related to vaccine quality remain prevalent. However, the emerging technology of differentiating DCs from human pluripotent stem cells (hPSC) holds great promise in resolving these challenges and unlocking the potential for large-scale production of DC vaccines in the future [19]. This breakthrough will enable a reliable source of DCs for continued enhancement of vaccine quality.

### Nucleic acid vaccines

In contrast to traditional protein-based vaccines, DNA vaccines offer a unique approach to immunization, harnessing the power of bacterial plasmids to encode functional genes [22,23]. These vaccines work by generating antigens within the body’s own cells, which are then presented by the major histocompatibility complex (MHC) through a specialized antigen presentation pathway. This activates T cells and triggers an immune response [24]. While most DNA vaccines currently in development focus on tumor-associated antigens (TSA), the clinical efficacy of such vaccines has been limited by immune tolerance [25]. To overcome this challenge, researchers are turning to tumor neoantigens, which are ideal targets for DNA vaccine development due to their specificity. In preclinical studies, multiple DNA vaccines synthesized using neoantigens have demonstrated significant CD8+ T cell-associated antitumor responses in mouse models [26]. In a recent investigation, it was revealed that a DNA-designed, personalized neoantigen vaccine, skillfully presented by DC cells, was shown to elicit a potent immune response in a murine melanoma cell model, resulting in a noteworthy decrease in lung metastasis of the malignant neoplasm [27].

To further enhance the immunogenicity of DNA vaccines, researchers are exploring the use of adjuvants and improved vaccine delivery systems. One recent study developed a novel DNA vaccine technology called “pTOP” (plasmid to deliver T cell epitopes) [28], which encodes modified VSV-G (vesicular stomatitis virus glycoprotein). This technology allows for the insertion of antigenic epitopes, promoting tumor specificity and inducing anti-epitope T cell responses. The pTOP technology has been shown to effectively promote immune recognition and elicit higher CTL activity, making it a promising avenue for future research. In addition to their potential for tumor immunotherapy, DNA vaccines offer the advantages of convenient design, low cost, and long-term stability *in vivo* [29]. While challenges remain in achieving optimal immunogenicity, ongoing research in this field holds great promise for the development of effective and accessible vaccines against a range of diseases.

In 2017, a groundbreaking achievement was made in the field of personalized RNA vaccines for melanoma [30]. In a study involving 13 patients with stage III/IV melanoma, eight patients demonstrated a robust immune response to the vaccine, resulting in the maintenance of disease-free status without recurrence or progression. In the remaining five patients with relapsed melanoma, three of them showed promising responses to the vaccine, leading to objective clinical responses. Remarkably, one patient attained complete remission after being vaccinated with anti-PD-L1 therapy.

Compared to DNA vaccines, RNA vaccines are simpler to prepare. RNA molecules can be easily translated into antigens in the cytoplasm without the need to enter the nucleus or integrate into the genome, minimizing the risk of insertion mutations and ensuring relative safety. RNA vaccines are poised to revolutionize immunotherapy against tumors [31,32]. Currently, RNA vaccines are considered a compelling alternative to DNA vaccines, and numerous preclinical trials have been initiated to assess their safety and effectiveness based on tumor neoantigens. Four patients with metastatic gastrointestinal cancer were administered vaccination with neoantigen mRNA vaccines in one such trial, which elicited specific T cell immune responses [33]. However, objective clinical responses have yet to be fully realized, and the clinical efficacy of RNA vaccines requires further investigation. Researchers are exploring various avenues to improve the delivery strategy of mRNA vaccines. For instance, the neoantigen RNA vaccine mRNA-4157 encapsulated in lipid-based nanoparticle (LNP) was employed alone or in conjunction with pembrolizumab to treat various solid tumors, yielding notable clinical responses and excellent safety and tolerance [34].

### Synthetic long peptide vaccines

The synthetic long peptide (SLP) vaccine design addresses the limitations of traditional MHC-I combined with short peptide vaccines, by effectively presenting antigens while avoiding immune tolerance issues that may arise from the latter [35]. Professional antigen-presenting cells (APCs) process SLP *in vivo* through cross-presentation of MHC-I/MHC-II molecules [36], which not only enhances CD8+ T cell responses but also induce CD4+ T cell helper effects. Additionally, SLP can prolong the duration of epitope presentation in the antigen-draining lymph node, leading to improved immunogenicity [35].

In recent years, clinical trials of neoantigen synthetic vaccines have also shown promise. A melanoma-based trial utilizing a neoantigen vaccine found that tumor neoantigens could effectively activate T cells [37]. The neoantigen-specific T cell pool was observed to expand, causing a shift in the balance of the tumor immune microenvironment, thus strengthening tumor control. This is likely due to neoantigens being the most effective targets for anti-tumor immune responses. Neoantigen vaccines have also shown efficacy in some tumors that have an immunologically “cold” tumor microenvironment. Keskin et al. [38] conducted a phase I/Ib study where they designed a synthetic long peptide vaccine based on methylguanine methyltransferase (MGMT)-unmethylated glioblastoma patients who were not responsive to dexamethasone treatment. Results showed that there were circulating multifunctional neoantigen-specific CD4 and CD8 T cell responses, and tumor-infiltrating T cells showed an increase. In another phase I/IIa clinical trial [39], a neoantigen vaccine was designed based on an immunogenic frameshift peptide (FSP) produced by DNA mismatch repair deficiency(dMMR), and was found to be systemically well-tolerated while consistently inducing humoral and cellular immune responses in dMMR colorectal cancer patients.

SLP vaccines are currently the leading research direction in clinical trials for neoantigen vaccine. Numerous studies have confirmed the safety and stability of SLP vaccines, providing significant advantages over other vaccine platforms. While synthetic peptides used alone may lack effective antigen presentation pathways, SLP vaccines have demonstrated strong potential when combined with immune adjuvants [40]. For instance, TLR3 agonist poly-ICLC [41] and STING agonist [42] have been shown to increase the efficacy of SLP vaccines. Despite the promising results obtained through the use of SLP vaccines in clinical trials, there is still a need for continuous efforts to achieve optimal immune response and effective anti-tumor activity. In summary, SLP vaccines have demonstrated promise as a cancer immunotherapy, and some application strategies have been summarized through clinical trials. Nevertheless, different types of vaccines have different ways of action (Fig. 1), further research is needed to optimize the immune response and anti-tumor activity of these vaccines.

### Multi-epitope vaccination

While neoantigens resulting from tumor-specific mutations are optimal targets for cancer immunization, high-throughput sequencing data derived from next-generation sequencing (NGS) technology has revealed that each individual tumor patient possesses dozens or even hundreds of distinct antigens. Consequently, it is challenging to implement a systemic targeted therapy approach that is predicated on a single epitope.

Currently, a plethora of multi-epitope neoantigen vaccines are progressively advancing towards the clinical trial phase, heralding a new era in the domain of immunotherapy. Castle et al. [43] employed NGS to detect all potential mutation sites in mouse melanoma and verified the immunogenicity of these sites by producing long peptide vaccines. Their study found that a considerable proportion of these neoantigens were immunogenic and were restricted by a large number of MHC-II molecules, which could be identified by CD4+ T cells and have an anti-tumor effect. Using bioinformatics technology, Kreiter et al. [44] rapidly screened and synthesized a multi-epitope neoantigen mRNA vaccine, which elicited an evident T cell immune response, based on the mutation level of exon sequencing and the ability to bind to MHC-II. Moreover, in another preclinical investigation, a multi-epitope DNA vaccine developed from neoantigens induced a potent immune response in a mouse model of breast cancer [45], and was able to elicit neoantigen-specific T cell responses in a subsequent patient with metastatic neuroendocrine tumors. Additionally, personalized, multi-epitope synthetic long peptide vaccines that were based on neoantigens were shown to induce neoantigen-specific T cells to migrate into the brain and improve the immune microenvironment of glioblastoma [38].

The recognition of tumors by the immune system is a complex process, involving multiple cell types and the identification of numerous epitopes. The recognition of tumor-associated epitopes by immune cells represents a critical step in anti-tumor immune responses. Notably, the complexity of tumor antigenicity is reflected in the involvement of a variety of immune cell populations, which work collaboratively to recognize and target multiple epitopes. In contrast to the traditional approach of designing vaccines targeting a single epitope, the development of personalized multi-epitope vaccines has emerged as a promising strategy with superior clinical application potential. Future research in this area holds great promise for the development of effective cancer immunotherapies.

## Neoantigen-Based Adoptive Cell Therapy

### Tumor infiltrating lymphocytes

**Figure 2:**
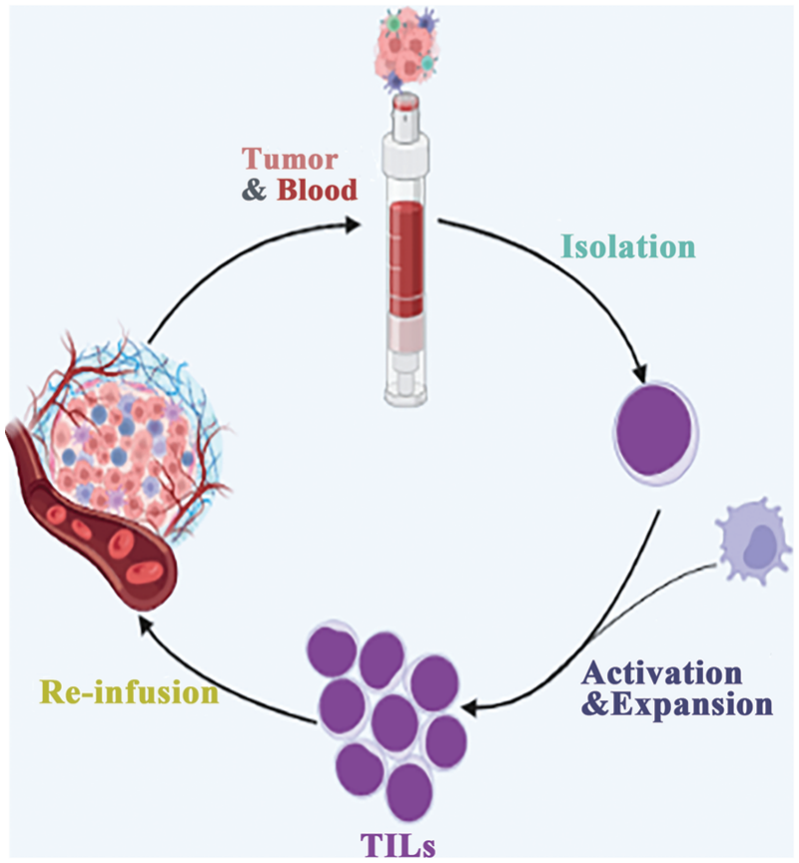
Direct infusion of *in vitro* expanded TILs.

Lymphocytes in the immune system play a critical role in identifying and eliminating tumor cells within the tumor microenvironment. Adoptive therapy, which involves *ex vivo* expansion of autologous tumor-infiltrating cells (TIL) (Fig. 2), has emerged as a promising approach. However, not all TILs are equally effective in immunotherapy. Recent studies have revealed that lymphocytes induced by specific somatic mutation epitopes on tumor cells exhibit better recognition and killing capabilities [46].

In a cohort of nine patients with metastatic gastrointestinal cancer [47], tumor-infiltrating lymphocytes were considered to be immunogenic to specific somatic mutation epitopes expressed by their own tumors. This discovery led to a series of studies on neoantigen-based TIL therapy. One study demonstrated that neoantigen-recognized CD8+ T cells can enhance TIL-mediated tumor killing [48]. Additionally, neoantigen-specific T cells were found to be associated with the success of TIL-ACT therapy. The stem cell-like neoantigen-specific TIL pool is retained in TIL-ACT responders [49], leading to its self-renewal and expansion *in vivo*, ultimately achieving anti-tumor effects.

However, identifying neoantigen-specific TILs remains a major challenge in current research. To overcome this problem, next-generation sequencing technology and bioinformatics are being utilized. Lowery et al. [50] employed single-cell transcriptomics to analyze the molecular characteristics of neoantigen-specific TILs and compared their characteristics. Based on the transcriptome status of TILs, T-cell receptors (TCRs) with neoantigen-specific recognition capabilities were predicted, thereby identifying neoantigen-specific TILs for tumor immunotherapy.

Overall, these findings provide a promising avenue for improving the efficacy of TIL therapy through the identification and expansion of neoantigen-specific TILs. Further research is needed to develop more efficient and effective methods of identifying these TILs, which can pave the way for more personalized and targeted immunotherapies against cancer.

### T cell receptor-gene engineered T cells

Direct modification of T cell receptor offers a more precise and effective approach compared to the direct reinfusion of tumor infiltrating lymphocytes expanded *in vitro*. Tumor-specific epitopes are critical in determining the phenotype and state of CD8+ T cells, with neoantigen-specific TCR (neoTCR) exhibiting higher affinity [51]. By genetically engineering TCR, it is possible to replace the endogenous TCR and transfer it into T cells, resulting in precise targeting of cells expressing tumor-specific antigens [52]. In a recent study, the potential of modified TCR for adoptive therapy was investigated in a cohort of seven patients with ovarian cancer [53], this therapeutic approach holds promise for the treatment of this malignancy. These findings provide preliminary evidence of the feasibility and effectiveness of the modified TCRs for adoptive therapy in ovarian cancer.

NeoTCR is the central focus of TCR-T cell therapy. In a clinical study, personalized neoantigen TCR-T therapy designed based on naturally occurring T cell receptors that recognize tumor-specific antigens was found to be effective against xenograft tumors [54]. The therapy had a remarkable therapeutic effect on patients, providing a solution to the problem of the source of neoantigen-specific recognition TCR modification. In addition, a cloning and expression system developed by Hu et al. [55] allows the assembly of any TCR and can be used for activation, enabling the quick evaluation of the specificity of TCR for neoantigen recognition and affinity for tumors. The recently developed CRISPR-Cas9-based technology can simultaneously knock out endogenous TCR and knock in neoTCR [56], leading to the design of a more personalized neoantigen TCR-T treatment plan. These studies have successfully preliminarily elucidated the entire process of neoTCR, starting from its source and identification, all the way to the integration of T cells. Further research is eagerly anticipated developing a comprehensive personalized treatment strategy.

### Chimeric antibody receptor engineered T cell

**Figure 3:**
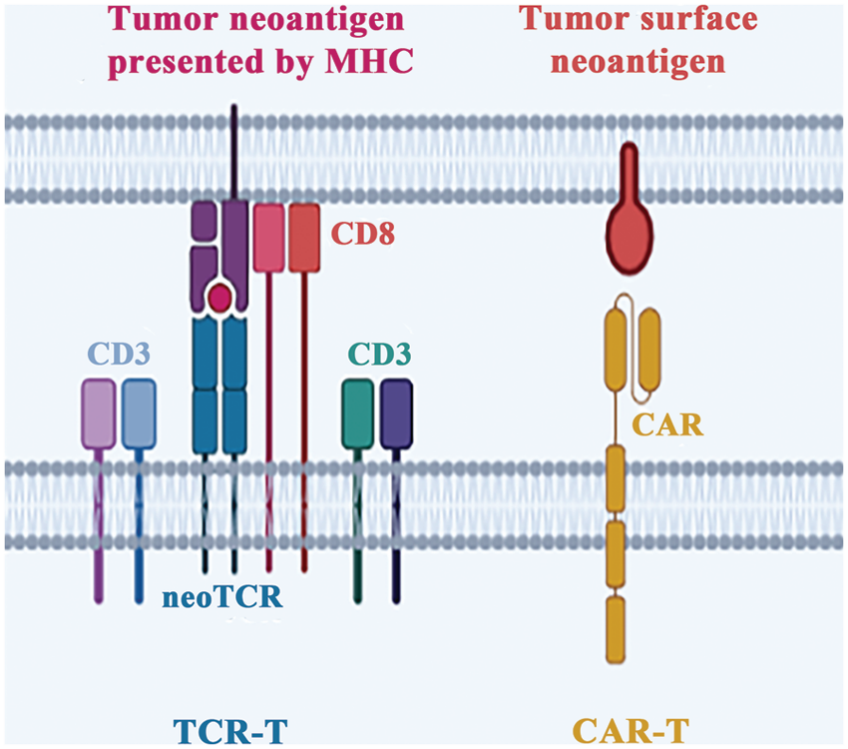
Different strategies of adoptive cell therapy. There are two primary methods to engineer T cells *in vitro*. The first involves equipping T cells with neoantigen-specific TCR (neoTCR), called TCR-T. The second approach entails replacing the original TCR with a chimeric antigen receptor and called CAR-T.

Chimeric Antibody Receptor Engineered T Cell (CAR-T) possess a distinct advantage over TCR-T cells in their ability to recognize antigens. Unlike TCR-T cells, which rely on HLA recognition and antigen presentation, CAR-T cells generate antigen-antibody interaction by directly binding to cell surface antigens through their extracellular antigen recognition domains (Fig. 3). This property makes them highly effective in the immunotherapy of tumors with low mutational burden and insufficient presentation of neoantigens [57]. However, a significant limitation of CAR-T cells is their ability to recognize only a limited number of tumor cell surface antigens, which restricts their clinical application. To overcome this limitation, researchers have explored the use of tumor neoantigens, which are generated as a result of tumor-specific mutations, as targets for CAR-T cells. For instance, CAR-T therapy developed based on the neoantigen epitope generated by the EGFRvIII mutation of glioblastoma has been found to effectively control tumor growth in clinical trials [58].

Despite the promise of CAR-T cells in cancer immunotherapy, their efficacy in solid tumors remains limited due to tumor heterogeneity [59], CAR-T cells cannot effectively recognize the specific antigens on the surface of tumors. In response, researchers have developed CAR-T technology based on the combination of multiple antigens designed by Boolean logic gates [60,61]. These circuits activate the specific recognition and killing effect of tumor neoantigens through logic circuits, which can be adjusted to overcome tumor heterogeneity and produce long-lasting and effective effects. Another promising approach involves using CRISPR-Cas9 to generate non-virus-specific targeting CAR-T cells [62]. This technology has shown good safety and feasibility in clinical trials, and CAR-T cells administered at low doses and concentrations have been found to be effective in cancer treatment.

In summary, CAR-T cells represent a promising approach in cancer immunotherapy, particularly in the treatment of tumors with low mutational burden and insufficient presentation of neoantigens. The use of tumor neoantigens and the development of Boolean logic gates and CRISPR-Cas9-based technologies have expanded the potential of CAR-T cells in treating solid tumors and overcoming tumor heterogeneity.

## Tumor Neoantigen Burden in Immunotherapy

### Neoantigen prediction

Similar to tumor mutation burden (TMB), neoantigen mutation burden (TNB) is defined by the number of neoantigen mutations present in megabases of a genomic region [63]. Prior to using immunogenic neoantigens for therapeutic purpose, it is necessary to predict their presence. Traditional methods of neoantigen detection can be inefficient and expensive. However, recent advances in bioinformatics have led to the development of a range of one-stop neoantigen prediction tools such as Pvac-Seq [64], eopepsee [65], TSNAD [66], CloudNeo [67], TIminer [68], and MuPeXI [69]. They work by predicting the binding affinity of peptides containing mutated amino acids to MHC molecules, targeting neoantigens derived from SNVs and small frameshift mutations. In addition, we also have the neoantigen prediction tool INTEGRATE-neo [70] for gene fusion sources, and the neoantigen prediction tool neoantigenR [71] for transcript alternative splicing sources.

Although the use of these tools improves the accuracy of neoantigen prediction, the *in vivo* efficacy of neoantigens is ultimately dependent on the TCR, and it is difficult to predict this through simple machine learning [72]. As a result, neoantigens still face numerous bioinformatics and clinical challenges before they can be fully utilized in the field of immunotherapy [73]. To overcome these challenges and establish a unified and effective algorithm standard for neoantigen prediction, multiple institutions and organizations have formed a global alliance [74]. This alliance aims to compare the prediction results of different methods on the same batch of data, integrate and improve the strength of each method, and gain insight into the immunomodulatory pathways involved. The adoption of this approach is expected to significantly advance the field of neoantigen prediction [75], unravel the mechanisms through which neoantigens regulate HLA molecules to exert immunogenicity, and promote the use of immunotherapy targeting neoantigens.

### Tumor neoantigen burden as a biomarker for immunotherapy

In recent years, immunotherapy has emerged as a promising approach for the treatment of various types of solid tumors. However, identifying patients who are most likely to benefit from immunotherapy remains a challenge. TMB has been proposed as a potential biomarker of immunotherapy response [76,77], as high TMB can increase the production of neoantigens, which can activate the immune system and enhance the killing ability of tumor cells. However, the use of TMB alone as a biomarker has been challenged [78–80], not all solid tumors are suitable for TMB analysis [81], such as breast cancer, prostate cancer, and glioma.

Recent studies have shown that TNB, which directly evaluates the number of neoantigens in tumors, may serve as a more effective biomarker of immunotherapy response in certain solid tumors. TNB has been found to be positively correlated with TMB, and can promote the activation of lymphocytes and improve the immune response to tumor cells. In particular, TNB-H has been found to effectively stimulate lymphocyte infiltration and the formation of an inflammatory environment in the early stage of tumorigenesis in dMMR colorectal cancer, before the TMB-H environment is formed [82]. In MSI colorectal cancer, neoantigens can promote the infiltration of TILs and enhance the response of CD8+ T cells [83], thus serving as the basis for personalized immunotherapy. Moreover, neoantigen burden has been found to be associated with the expression of immune-related genes, such as M1-type polarized macrophage genes, PD-1, PD-L1, IFNγ, GZMB, and FASLG, and may have potential value as a biomarker in lung cancer immunotherapy [84].

In conclusion, while TMB has been proposed as a biomarker of immunotherapy response, recent studies suggest that TNB may serve as a more effective biomarker for certain types of solid tumors. Further research is needed to determine the clinical utility of TNB as a biomarker for immunotherapy response and to identify the optimal cutoff values for different types of solid tumors.

### Tumor neoantigen burden predicts immunotherapy and other therapies efficacy

High TNB-generated neoantigens have been shown to potentially trigger a more effective tumor immune response and play a greater role in immunotherapy. Therefore, the use of TNB as a predictive tool for the clinical prognosis of immunotherapy is of great interest. In a study of patients with advanced melanoma [85], a prognostic model established by the TNB score combined with immune-related drug resistance mechanisms was found to more accurately forecast the prognostic response to immunotherapy than other predictive models.

Previous studies have suggested that some cancer patients can enhance the effect of immunotherapy after radiotherapy or chemotherapy, in part due to the increase in neoantigen exposure [86]. In patients with metastatic NSCLC, for example, radiotherapy was found to induce an enhanced blocking effect of anti-CTLA-4 antibody and induce an anti-tumor T cell response throughout the body [87]. Additionally, radiation can change the immune status of irradiated tumor cells, transforming them into an effective native vaccine with strong immunogenicity [88].

Similarly, after receiving neoadjuvant chemotherapy, patients with locally advanced colorectal cancer developed new mutations and enhanced the efficacy of anti-PD-1, possibly due to the increased neoantigen load [89]. The relationship between neoantigen load and the prognosis of clinical treatment has also been observed in breast cancer [90], ovarian cancer [91], melanoma [92], multiple myeloma [93], and other cancers.

While the clinical application of TNB is not yet perfect, further research is needed to provide more accurate and effective clinical treatment guidance for patients through comprehensive evaluation combined with other indicators.

## Combination Therapy Based on Neoantigen Therapy

### Neoantigen vaccines in combination with other treatments

The complexity of immune heterogeneity in tumors, coupled with the evolving immune evasion mechanisms of tumor cells [94], limits the efficacy of single immunosuppressant therapy. To achieve a strong combination effect, immunotherapy with multiple mechanisms and immune targeted therapy at different stages of tumor development can be combined [7,95]. The commonly used immunosuppressant targets PD-1/PD-L1 and CTLA-4 can inhibit the killing effect of CTL on tumor cells, while neoantigen vaccines can activate immune cells in the tumor microenvironment. Combining the two can produce a more significant anti-tumor effect.

A multi-epitope RNA vaccine designed based on neoantigens produced a complete response to combined PD-1 blockade therapy in a melanoma patient [30]. In another trial, the neoantigen vaccine expanded the pre-existing neoantigen-specific T cell population, and two patients had complete tumor regression after receiving anti-PD-1 therapy [37]. The combination of neoantigens and immune checkpoint inhibitors can improve the immune microenvironment in tumors, inducing CD8+ T cells to control tumors [96].

Radiotherapy and chemotherapy can increase the release of neoantigens [86,89,97], improve the immune microenvironment, and enhance T cell responses, showing potential for combined immunotherapy [98]. DC vaccines have also been shown to be safe and feasible in combination with chemotherapy in glioblastoma [99].

In conclusion, combining various mechanisms of therapy and immune targeted therapy at different stages of tumor development can lead to a strong combination effect. Combining neoantigen vaccines with immune checkpoint inhibitors, radiotherapy, or chemotherapy can improve the immune microenvironment, enhance T cell responses, and induce CD8+ T cells to control tumors. These findings provide new directions for combining immunotherapy in the treatment of tumors.

### Neoantigen-based ACT in combination with other treatments

Combinatorial approaches are leveraging neoantigen vaccine therapies, in conjunction with ACT and other immune-targeting modalities such as PD-1/PD-L1 blockade, have emerged as a promising avenue for enhancing the clinical efficacy of cancer treatments (Table 1). By bolstering the immune environment through immunotherapies, as well as conventional radiotherapy and chemotherapy, it is possible to generate long-lived memory T cells with high persistence and self-renewal capability [100]. This approach enhances the maintenance effect of CAR-T and promotes their sustained anti-tumor activity.

**Table 1:** Partially recruited or completed clinical trials of neoantigen combination therapy

Platform	Phase	Cancer type	Combinnd therapies	ClinicalTrial.gov identifier
DC vaccine	II	Hepatocellular Cancer Colorectal Cancer	Nivolumab	NCT04912765
DNA vaccine	I/II	Hepatocellular Cancer	NO-9012 Pembrolizumab	NCT04251117
	II	Small Cell Lung Cancer	Durvalumab	NCT04397003
mRNA vaccine	/	Gastric Cancer Esophageal Cancer Liver Cancer	PD-1/L1	NCT05192460
SLP vaccine	I	Pancreatic Cancer Metastatic Colorectal Cancer Metastatic	Retifanlimab	NCT04799431
	I/II	Melanoma Ocular Melanoma Uveal Melanoma	6MHP PolyICLC CDX-1140	NCT04364230
	I	Squamous Cell Lung Cancer Squamous Non-small Cell Lung Cancer Squamous Cell Carcinoma of Head and Neck	Pembrolizumab	NCT04266730
TIL	I	Epithelial Tumors	NMA-LD Regimen Interleukin-2	NCT05141474
TCR-T	II	Endocrine Tumors Non-Small Cell Lung Cancer Ovarian Cancer Breast Cancer Gastrointestinal/Genitourinary Cancers Neuroendocrine Tumors Multiple Myeloma	Cyclophosphamide Fludarabine Aldesleukin Pembrolizumab	NCT03412877
	II	Non-Small Cell Lung Cancer Breast Cancer Gastrointestinal/Genitourinary Cancers Ovarian Cancer	Fludarabine Cyclophosphamide Aldesleukin	NCT04102436
CAR-T	I	Glioblastoma	Pembrolizumab	NCT03726515

Studies have shown that neoantigen vaccination can promote the generation of T cells, resulting in increased numbers of neoantigen-specific T cells in circulation [30,101]. Furthermore, neoantigen vaccination promotes the diversification of the neoTCR pool, thereby improving the therapeutic effect of ACT [102]. A vaccine for enhancing the therapeutic effect of CAR-T has been designed [103], and direct injection of amphiphilic CAR-T ligands (amph-ligands) to target lymphocytes can initiate and enhance CAR-T function.

Immunotherapies developed based on neoantigens have been shown to hold promise in combination [104]. This approach has the potential to overcome limitations of current monotherapies by augmenting the immune response against cancer and promoting sustained therapeutic activity. Further research and development in this area may yield novel immunotherapeutic approaches with increased efficacy in cancer treatment.

### Exploring the potential of combined therapies

Identification and targeting of neoantigens, peptides derived from tumor-specific mutations, have emerged as a promising strategy for cancer immunotherapy. However, screening and validating neoantigens from a large pool of mutated tumor cells remain challenging. An alternative approach worth considering is to actively induce the expression of neoantigens using specific methods.

Recent evidence suggests that neoadjuvant chemotherapy followed by immunotherapy can effectively transform the tumor microenvironment, leading to an increased probability of tumor mutations and higher tumor mutational burden (TMB) [86,97]. As TMB rises, the exposure of neoantigens also increases, which could be leveraged for multi-epitope neoantigen immunotherapy through radiotherapy. Stereotactic Body Radiation Therapy (SBRT), a precision radiation technique, has shown superior efficacy with reduced normal tissue damage compared to conventional radiotherapy. In addition to its direct cytotoxic effect on tumor cells, SBRT can improve the tumor immune microenvironment, normalize tumor blood vessels, and synergism with traditional immunotherapy [105,106].

By inducing precise high-dose irradiation to tumors, SBRT can generate or expose more neoantigens, which could be further combined with various neoantigen-based therapies for enhancing efficacy [107]. This field holds great potential for innovative approaches to cancer immunotherapy and merits further exploration.

## Prospect

In the realm of immunotherapy, a myriad of challenges has arisen, impeding the progress of this groundbreaking approach. Delving deeper into the intricacies of its treatment mechanism, a disheartening realization was uncovered: immunotherapy does not cater to everyone. A case in point would be the efficacy of anti-PD-1/PD-L1 therapy, which is significantly reduced in pMMR colorectal cancer patients compared to their dMMR counterparts. However, amidst these obstacles, lies an opportunity to transcend the current limitations of immunotherapy. By crafting neoantigen immunotherapies, a path to a breakthrough may be paved.

Due to their uniqueness and natural origin, neoantigens provide powerful targets for tumor immunotherapy and play a significant role in the field of cancer immunotherapy. Immunotherapies based on neoantigens have great feasibility. This article summarizes the current clinical progress of tumor neoantigens, mainly including tumor vaccines based on neoantigens: DC vaccines, nucleic acid vaccines, and synthetic long-peptide vaccines, as well as adoptive cell therapy: TILs, TCR-T, and CAR-T. In addition, the TNB improved based on the TMB is a valuable biomarker in immunotherapy, and is expected to serve as a powerful factor in predicting the efficacy and prognosis of immunotherapy. In addition to the prediction method of neoantigen need future improved, in-depth research is also needed in various application methods of neoantigens with regard to their immune mechanisms. Currently, most treatment modalities based on neoantigens remain under development. No clear advantages or disadvantages exist among the different application pathways within the current research landscape. However, they exhibit varying applications in different tumor types of due to tumor heterogeneity. In clinical settings, practical applications face numerous ethical dilemmas due to the significant lack of effective patient recruitment and insufficient support systems. Personalized neoantigen tumor treatment lacks a complete set of guidelines from sample collection to the individual patient application to protect patient rights and achieve better treatment benefits. From a theoretical stage standpoint, present experimental technologies and theories require further refinement, and most developmental applications necessitate extensive time to address efficiency and economic concerns.

The two primary clinical applications of neoantigens are tumor vaccines and adoptive cell therapy, both of which exert immunotherapeutic effects by identifying and targeting neoantigens. Leveraging advancements in artificial intelligence (AI) and molecular mechanisms research of the tumor immune microenvironment, the future holds immense potential. Employing continuously optimized machine learning algorithms to calculate and accurately identify immunogenic neoantigens and their cognate TCRs, in conjunction with a deeper comprehension of tumor immune microenvironment molecular mechanisms, may facilitate the discovery of an optimal neoantigen action pathway. Such pathways could include devising the ideal neoantigen treatment strategy and establishing a modular neoantigen treatment pathway, potentially accelerating the clinical application of neoantigens.

The future of neoantigen therapy envisions AI-assisted algorithms rapidly and precisely recognizing immunogenic neoantigens and cognate TCRs in individual tumor patients. By simply replacing antigens targeting distinct tumors, it may be possible to design tumor vaccines or neoTCRs with heightened neoantigen recognition thereby efficiently conducting tumor immunotherapy. As such, considerable advancements in neoantigens are anticipated in the future.
